# Differential associations of central and brachial blood pressure with carotid atherosclerosis and microvascular complications in patients with type 2 diabetes

**DOI:** 10.1186/1471-2261-14-23

**Published:** 2014-02-20

**Authors:** Chan-Hee Jung, Sang-Hee Jung, Kyu-Jin Kim, Bo-Yeon Kim, Chul-Hee Kim, Sung-Koo Kang, Ji-Oh Mok

**Affiliations:** 1 Division of Endocrinology and Metabolism, Department of Internal Medicine, Soonchunhyang University College of Medicine, #170 Jomaru-ro, Wonmi-gu, Bucheon-si, Gyeonggi-do 420-767, South Korea; 2 Department of Obstetrics and Gynecology, Cha University School of Medicine, Bundang, Korea

**Keywords:** Central blood pressure, Brachial blood pressure, Microvascular complications, Carotid atherosclerosis, Type 2 diabetes

## Abstract

**Background:**

We examined the relationship between central blood pressure (BP), brachial BP with carotid atherosclerosis and microvascular complications in type 2 diabetes mellitus (T2DM).

**Methods:**

We recruited 201 patients who were evaluated for central BP, brachial BP, carotid ultrasonography, brachial-ankle pulse wave velocity (baPWV), ankle-brachial index (ABI) and microvascular complications. Central BP were calculated using a radial automated tonometric system.

**Results:**

Agreement between central BP and brachial BP was very strong (concordance correlation coefficient between central and brachial SBP = 0.889, between central and brachial PP = 0.816). Central pulse pressure (PP) was correlated with mean carotid intima-media thickness (CIMT), baPWV and ABI, whereas brachial PP was borderline significantly correlated with CIMT. The prevalence of nephropathy(DN) and retinopathy(DR) according to the brachial PP tertiles increased, the prevalences of microvascular complications were not different across central PP tertiles. In multivariate analysis, the relative risks (RRs) for the presence of DR were 1.2 and 4.6 for the brachial PP tertiles 2 and 3 when compared with the first tertile. Also, the RRs for the presence of DN were 1.02 and 3 for the brachial PP tertiles 2 and 3 when compared with the first tertile.

**Conclusions:**

Agreement of central BP and brachial BP was very strong. Nonetheless, this study showed that higher brachial PP levels are associated with increased probability for the presence of microvascular complications such as DR/DN. However, there are no associations with central SBP and central PP with microvascular complications. Central BP levels than brachial BP are correlated with surrogate marker of macrovascular complications.

## Background

Blood pressure (BP) management is important for the prevention and management of cardiovascular disease (CVD) and microvascular complications in T2DM [1]. Brachial BP remains the standard of reference for the evaluation and management of BP, and has been a key element in predicting target organ damage (TOD) and CVD [2]. However, there is increasing evidence that central BP may be a more sensitive indicator of CV risk than brachial BP in specific groups [3-5]. In a study of American Indians, central BP more strongly related to the extent of carotid atherosclerosis, vascular hypertrophy and CV events than brachial BP [4]. The Conduit Artery Function Evaluation study demonstrated the superiority of central BP to brachial BP as a CV predictor in hypertensive patients [5]. In patients with T2DM, a few studies have documented that increased augmentation of central BP is associated with increases in CIMT [6,7]. However, to our knowledge, no study has compared central BP with brachial BP regarding to association with both micro-and macrovascular complications in patients with T2DM.

Pulse pressure (PP) is traditionally thought of as a marker of arterial stiffness and has been suggested as an independent CV risk factor [8,9]. Recent several studies reported that brachial PP may be significantly associated with CIMT [10,11]. Brachial PP is reportedly a better predictor of coronary heart disease events than other BP components in patients with T2DM [9]. However, the significance of central PP versus brachial PP regarding macrovascular complications in patients with diabetes remains to be clarified. In addition, some authors suggested that brachial PP is associated with microvascular complications, although some authors disagree [12-14].

Central BP is most accurately measured by an invasive method. It has been evaluated noninvasively by mathematically transforming the radial artery pulse waveform to the aortic pulse waveform recently [15,16]. Although a few studies evaluated the relations of brachial and central pressures to carotid atherosclerosis, no studies have reported the relative importance of central and brachial BP in to microvascular complications in patients with T2DM.

Therefore, the aim of this study was to evaluate the value of central BP and brachial BP components in relation to microvascular complications and surrogate markers of macrovascular diseases (CIMT, baPWV and ABI), in patients with T2DM.

## Methods

### Patients

We recruited 201 patients with T2DM who were evaluated for central BP, carotid ultrasonography and standard brachial BP measurement at the diabetes clinic of Soonchunhyang University Bucheon Hospital, from June 2012 to July 2012. We reviewed detailed demographic data, biochemical data and clinical history using medical records. Participants provided written informed consent for the use of their data for research. This study was reviewed and approved by the Institutional Review Board of Soonchunhyang University College Medicine, Bucheon Hospital.

### Measurement of central BP

Central BP was evaluated noninvasively by mathematically transforming the radial artery pulse waveform to the aortic pulse waveform with an automated tonometric system, HEM-9000AI (Omron Healthcare, Kyoto, Japan) in a sitting position after at least 5 min of rest. The radial artery pressure waveform was recorded for 10 sec with the HEM-9000AI system. The radial pulse wave was calibrated to brachial BP, measured with an automated oscillometric device. From the average radial pulse wave form, the corresponding ascending aortic pulse wave form was derived, using a validated generalized transfer function incorporated in the software (Omron Healthcare), which also provided the calculated central BP and the calculated central Aix. The measurements of blood pressure were performed twice by the same trained observer in same day at intervals of at least one minute.

### Carotid atherosclerosis

Carotid atherosclerosis was assessed by the use of a model SSA-660A high-resolution B-mode ultrasonograph device (Toshiba, Tokyo, Japan) performed with an ultrasound scanner equipped with a 12-MHz linear-array transducer. IMT measurements were performed on the right and left common carotid arteries 1.0 cm proximal to the origin of the bulb and the mean IMT values were calculated. Carotid IMT thickening was defined as mean CIMT ≥ 1.0 mm [17,18].

### Microvascular complications

Diabetic nephropathy (DN) was defined using albuminuria, which was measured by radioimmunoassay (Immunotech, Prague, Czech Republic). Albumin excretion rate (AER) in the range of 20-200 μg/min or urine albumin 30-300 mg/g creatinine was defined as microalbuminuria, and AER > 200 μg/min or urine albumin ≥ 300 mg/g creatinine as overt proteinuria. Patients were considered to have nephropathy if they displayed microalbuminuria or overt proteinuria.

Diabetic retinopathy (DR) was evaluated by experienced ophthalmologists while the patients’ pupils were dilated. If needed, fluorescein angiography was performed. DR was classified as normal, nonproliferative and proliferative retinopathy [19]. Patients were considered to have retinopathy if they displayed the nonproliferative or proliferative stage.

Diabetic peripheral neuropathy (DPN) was diagnosed with recommendation by the Expert Committee of Korean Diabetes Neuropathy Study Group, as: the presence of typical symptoms using the Michigan Neuropathy Screening Instrument (MNSI) and compatible findings on neurologic screening examinations or electrophysiologic studies [20,21]. Although electrophysiological studies are not essential, current perception threshold (CPT) test was performed in all patients using a Neurometer CPT/C (Neurotron, Baltimore, MD).

Cardiac autonomic neuropathy (CAN) was assessed by autonominc function test (AFT). CAN was assessed by the five standard cardiovascular reflex tests according to the Ewing’s protocol [22]. The severity of CAN was quantitated by summation of points obtained from each of the five tests, where each test was given a point of 0, 0.5, or 1 if it yielded a normal, borderline, or abnormal value, respectively. CAN was defined as the presence of at least two abnormal tests or an autonomic neuropathy points ≥ 2 [23,24].

An automated device (VP-1000; Colin, Komaki, Japan) was used to measure arterial baPWV and ABI. The insulin resistance status was evaluated by the HOMA-IR index, which was calculated by the formula: [fasting insulin (uIU/mL) × fasting blood glucose (mmol/L)]/22.5. The HOMA-IR score was available only in 164 patients not receiving exogenous insulin.

### Statistical analyses

Data are presented as mean ± standard deviation (SD) for variables normally distributed or as median (interquartile range) for variables not normally distributed or as number of participants (percentages). Non-normally distributed variables of, triglyceride, high-sensitivity C-reactive proein (hsCRP) and HOMA-IR were transformed as natural logarithm before analysis. The concordance correlation coefficient between central BP and brachial BP was measured to evaluate the agreement between two variables. The categorical variables of the groups were compared by Chi-square test. Correlation between BP and other clinical parameters were analyzed by Spearman’s correlation analysis. The significance of the mean differences including several parameters of BP between patients with and those without microvascular complications was evaluated with Student’s t-test. Patients were divided into theree groups by the tertiles of central or brachial PP levels, respectively. One-way ANOVA was used to evaluate differences of means among tertiles of central or brachial PP groups. The prevalence of microvascular complications and carotid atherosclerosis according to the tertile of brachial PP, central SBP and central PP were analyzed using Chi-square test. Relationships of central BP and brachial BP with microvascular complications were determined in multivariate logistic regression analyses. Two-tailed p < 0.05 was considered significant. Statistical analyses were performed with SPSS, version 18 (SPSS, Chicago, IL).

## Results

### General characteristics of the study populations

A total of 201 patients with T2DM (115 males) participated in this cross-sectional study. Clinical and biochemical characteristics of the study subjects are presented in Table 1. The mean age was 55.8 years and duration of DM was 8.5 years. Ninety-six (47.8% of total, 57% of men and 43% of women) were treated for hypertension. Eighty-three (86.5%) were treated with angiotensin converting enzyme inhibitor (ACEI) or/and angiotensin receptor blocker (ARB), 41 (47.2%) were treated with calcium channel blocker, 14 (14.6%) with beta blockers and 15 (15.6%) with diuretics. Central and brachial blood pressures and parameters of carotid atherosclerosis are presented in Table 1. The prevalence of DN, DR, DPN and CAN was 23.4%, 19.4%, 28% and 32.6%, respectively.

**Table 1:** General characteristics of the study populations

**Age (year)**	**55.8 ± 11.3**
Men/Women (%)	115/86 (57.2/42.8)
Duration of DM(year)	8.5 ± 7.5
Hypertension, n (%)	96 (47.8%)
Body mass index (kg/m^2^)	24.9 ± 3.1
Central SBP (mmHg)	121.7 ± 17
Central PP (mmHg)	49.4 ± 13.3
Brachial PP (mmHg)	49 ±12
Systolic BP (mmHg)	121.4 ± 15.6
Diastolic BP (mmHg)	72.3 ± 10
Total Cholesterol (mg/dL)	162.7 ± 36.2
Triglyceride (mg/dL)*	121 (88, 169)
HDL-cholesterol (mg/dL)	48 ± 13
LDL-cholesterol (mg/dL)	94.7 ± 33
hsCRP (mg/dL)*	0.09 (0.05, 0.18)
HbA1_C_ (%)	7.6 ± 1.6
eGFR (mL/min/1.73 m^2^)	76 ± 18
Mean CIMT (mm)	0.62 ± 0.14
Mean ABI	1.14 ± 0.07
Mean baPWV (cm/sec)	1555 ± 405
HOMA-IR*	2.81 (1.89, 4.5)
DPN, n(%)	56 (28%)
CAN, n (%)	65 (32.6%)
Diabetic nephropathy, n (%)	47 (23.4%)
Diabetic retinopathy, n (%)	39 (19.4%)

Data are reported as mean ± standard deviation (SD) for variables which are normally distributed or as median (interquartile range) for variables which are not normally distributed or as number of participants (percentages). DM: diabetes mellitus; SBP: systolic blood pressure; PP: pulse pressure; HDL: high density lipoprotein; LDL: low density lipoprotein; hsCRP: high-sensitivity C-reactive protein; HbA1c: hemoglobin A1c; eGFR: estimated glomerular filtration rate; CIMT: carotid intima-media thickness; ABI: ankle-brachial index; baPWV: brachial-ankle pulse wave velocity; HOMA-IR: homeostasis model assessment-insulin resistance; DPN: diabetic peripheral neuropathy; CAN: cardiac autonomic neuropathy.

*Natural logarithmic transformation were performed before analysis.

### Bivariate correlations between central and brachial BP with carotid atherosclerosis, vascular stiffness and clinical CV risk factors

**Figure 1:**
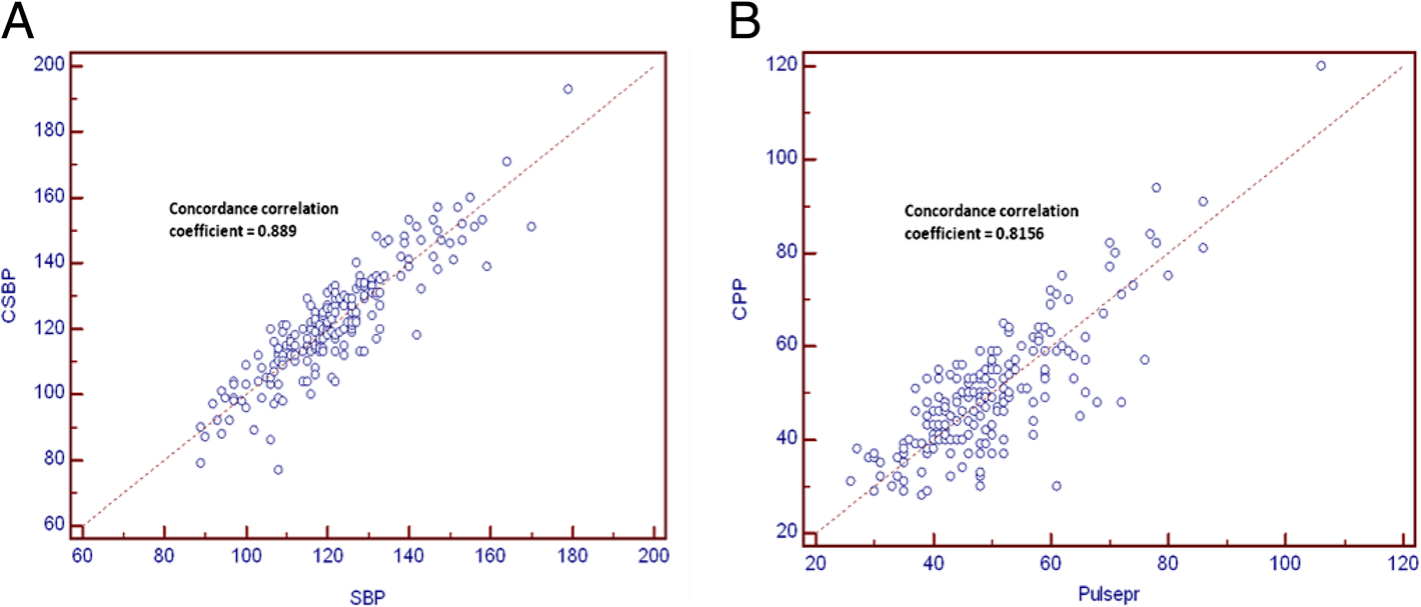
**Concordance correlation coefficient between central BP and brachial BP components. (A)** Concordance correlation between central SBP and brachial SBP. **(B)** Concordance correlation between central PP and brachial PP.

The concordance correlation coefficient between central SBP and brachial SBP was 0.889 and between central PP and brachial PP was 0.816 (Figure 1).

The correlations of central BP components (central SBP, central PP) and brachial BP components (brachial SBP, brachial PP) to carotid atherosclerosis, vascular stiffness and other clinical variables are presented in Table 2. Central SBP showed significant positive correlation with mean ABI and baPWV (r = 0.162, p = 0.04, r = 0.449, p < 0.01, respectively). Brachial SBP levels were correlated positively with total cholesterol, triglyceride and baPWV and were not correlated ABI. Brachial DBP showed negative correlations with age and duration of DM and positive correlations with eGFR and triglyceride (data not shown). Central PP was correlated positively with age, ABI and baPWV and negatively with eGFR. Brachial PP was not correlated with ABI and was correlated positively with duration of DM, age, eGFR, and baPWV. (r = 0.234, p < 0.01 for duration of DM; r = 0.473, p < 0.01 for age; r = −0.349, p < 0.01 for eGFR; r = 0.576, p < 0.01 for baPWV). Univariate analysis revealed that central PP is significantly correlated with CIMT (r = 0.235, p = 0.01) and brachial PP values also showed positive correlation but weaker correlation than central PP (r = 0.197, p = 0.047).

**Table 2:** Bivariate correlations between central and brachial blood pressure with clinical variables

	**Central SBP**		**Central PP**		**Brachial SBP**		**Brachial PP**	
	**r**	**p**	**r**	**p**	**r**	**p**	**r**	**p**
Age (years)	0.131	0.06	0.431	<0.001	0.119	0.091	0.473	<0.01
Duration of DM (years)	0.019	0.78	0.126	0.083	0.082	0.26	0.234	<0.01
BMI (kg/m^2^)	0.049	0.49	−0.015	0.834	0.091	0.2	0.04	0.56
Central SBP (mmHg)	-	-	0.767	<0.001	0.875	<0.001	0.544	<0.01
Central PP (mmHg)	0.767	<0.001	-	-	0.596	<0.001	0.748	<0.001
Brachial SBP (mmHg)	0.875	<0.01	0.596	<0.001	-	-	0.692	<0.01
Brachial DBP (mmHg)	0.643	<0.01	0.077	0.274	0.66	<0.001	0.005	0.94
Brachial PP (mmHg)	0.643	<0.01	0.748	<0.001	0.692	<0.001	-	-
HbA1_C_ (%)	0.06	0.4	−0.039	0.5	0.042	0.561	−0.034	0.64
eGFR (mL/min/1.73 m^2^)	−0.083	0.29	−0.217	0.006	−0.152	0.459	−0.349	<0.01
Total cholesterol (mg/dL)	0.143	0.06	0.068	0.376	0.194	0.011	0.139	0.07
Triglyceride (mg/dL)	0.108	0.15	−0.057	0.451	0.164	0.029	0.016	0.84
HDL-C (mg/dL)	0.051	0.504	0.035	0.647	0.08	0.293	0.035	0.65
LDL-C (mg/dL)	0.121	0.16	0.049	0.565	0.127	0.138	0.066	0.44
HsCRP (mg/dL)	0.1	0.31	0.072	0.469	0.072	0.466	0.037	0.44
HOMA-IR	0.05	0.62	0.02	0.844	0.071	0.481	0.068	0.5
Mean CIMT (mm)	0.082	0.38	0.235	0.01	0.068	0.467	0.197	0.047
Mean ABI	0.162	0.04	0.185	<0.019	0.083	0.297	0.069	0.38
Mean baPWV (cm/sec)	0.449	<0.01	0.531	<0.001	0.477	<0.001	0.576	<0.01

DM: diabetes mellitus; BMI: body mass index; SBP: systolic blood pressure; PP: pulse pressure; HbA1c: hemoglobin A1c; eGFR: estimated glomerular filtration rate; HDL: high density lipoprotein; LDL: low density lipoprotein; hsCRP: high-sensitivity C-reactive protein; HOMA-IR: homeostasis model assessment-insulin resistance; CIMT: carotid intima-media thickness; ABI: ankle-brachial index; baPWV: brachial-ankle pulse wave velocity.

Spearman’s correlation analysis was used for the statistical analyses.

### Carotid atherosclerosis, vascular stiffness and other clinical variables according to the tertile of central PP and brachial PP

Comparison of carotid atherosclerosis, vascular stiffness and clinical variables among tertile groups of central PP or brachial PP is shown in Tables 3 and 4. The mean CIMT, baPWV and ABI was significantly increased progressively across central PP tertiles (p = 0.04, p < 0.001, and p = 0.023, respectively). In addition, the age and duration of DM were significantly increased progressively across central PP tertiles (Table 3). Stage of DN (normoalbuminuria, microalbuminuria, overt proteinuria) among tertile groups of central PP was not different (p = 0.69). Whereas stage of DN among tertile groups of brachial PP was significantly different (p = 0.02). The age and duration of DM were significantly increased progressively, eGFR were decreased progressively across brachial PP tertiles (Table 4). Mean CIMT and baPWV in the third tertile group of a brachial PP were significantly higher than those levels in the first and second tertile group (p = 0.03, p < 0.01). Mean carotid IMT levels among tertile groups of central SBP and brachial SBP were not different (data not shown).

**Table 3:** Difference of mean values of the clinical variables according to the tertile levels of central PP

	**1**^**st **^**tertile**	**2**^**nd **^**tertile**	**3**^**rd **^**tertile**	**P for trend**
Central PP (mmHg)	37.1 ± 4.2	48.6 ± 2.8	63.7 ± 10	0.001
Central SBP (mmHg)	108.2 ± 11.8	122.1 ± 10.4	135.9 ± 13.8	<0.01
Brachial SBP (mmHg)	111.4 ± 11.9	121.7 ± 11.4	132.0 ± 15.1	<0.01
Brachial DBP (mmHg)	71.1 ± 10	73.4 ± 9.8	72.2 ± 10.2	0.53
Brachial PP (mmHg)	40.3 ± 7.2	48.2 ± 7.3	59.7 ± 10.6	<0.01
Age (years)	51.2 ± 11	54.2 ± 10.3	59.7 ± 10.6	<0.01
BMI (kg/m^2^)	24.9 ± 3.4	25.1 ± 2.7	24.7 ± 3.2	0.67
Duration of DM (years)	7.5 ± 5.4	7.3 ± 6.4	10.5 ± 9.2	0.02
HbA1_C_ (%)	7.7 ± 1.6	7.5 ± 1.5	7.6 ± 1.9	0.75
Total cholesterol (mg/dL)	158.3 ± 34.6	164.6 ± 37.6	166 ± 36.7	0.27
Triglycerides (mg/dL)	112 (91, 169)	133 (88, 185)	114 (77, 144)	0.35
HDL-C (mg/dL)	47.5 ± 11.9	47.6 ± 13.2	48.5 ± 13.7	0.67
LDL-C (mg/dL)	93.5 ± 32.6	93.4 ± 34.8	98.9 ± 31.4	0.45
eGFR (mL/min/1.73 m^2^)	79.5 ± 16.1	78.5 ± 19	69.5 ± 16	0.01
Diabetic nephropathy, n (%)				0.69
No albuminuria	55 (36.9)	52 (34.8)	42 (28.2)	
Microalbuminuria	13 (35)	12 (31.6)	13 (44.4)	
Overt proteinuria	2 (26.8)	4 (34.2)	3 (33.3)	
HOMA-IR	2.6 (1.8, 4.5)	2.9 (2.0, 4.1)	2.9 (1.9, 5.0)	0.92
hsCRP (mg/dL)	0.09 (0.05, 0.13)	0.08 (0.04, 0.18)	0.1 (0.05,0.25)	0.35
Mean CIMT (mm)	0.58 ± 0.14	0.62 ± 0.13	0.66 ± 0.15	0.014
Mean baPWV (cm/sec)	1385 ± 253	1581 ± 495	1751 ± 359	<0.01
Mean ABI	1.13 ± 0.06	1.15 ± 0.06	1.15 ± 0.06	0.02

Data are shown as mean ± SD, median (interquartile range) or number (percentages).

PP: pulse pressure; SBP: systolic blood pressure; SBP: systolic blood pressure; DBP: diastolic blood pressure; BMI: body mass index; DM: diabetes mellitus; HbA1c: hemoglobin A1c; HDL: high density lipoprotein; LDL: low density lipoprotein; eGFR: estimated glomerular filtration rate; HOMA-IR: homeostasis model assessment-insulin resistance; hsCRP: high-sensitivity C-reactive protein; CIMT: carotid intima-media thickness; baPWV: brachial-ankle pulse wave velocity; ABI: ankle-brachial index.

**Table 4:** Difference of mean values of the clinical variables according to the tertile levels of brachial PP

	**1**^**ST **^**tertile**	**2**^**nd **^**tertile**	**3**^**rd **^**tertile**	**P for trend**
Central PP (mmHg)	40.1 ± 6.6	47.5 ± 7.0	60.6 ± 14.6	<0.01
Central SBP (mmHg)	113.2 ± 13	119.9 ± 12.3	132 ± 19.1	<0.01
Brachial SBP (mmHg)	110.9 ± 11.6	120 ± 9.8	133.5 ± 15.5	<0.01
Brachial DBP (mmHg)	73.1 ± 9.6	72.4 ± 9.4	71.3 ± 10.9	0.3
Brachial PP (mmHg)	37.8 ± 4.3	47.6 ± 2.1	62.1 ± 10.2	<0.01
Age (years)	50.5 ± 9	54.6 ± 10.7	62.4 ± 10.8	<0.01
BMI (kg/m^2^)	24.5 ± 3.2	25.5 ± 3	24.7 ± 3	0.82
Duration of DM (years)	6.1 ± 5.1	7.7 ± 5.7	11.8 ± 9.9	<0.01
HbA1_C_ (%)	7.8 ± 1.6	7.6 ± 1.8	7.5 ± 1.5	0.3
Total cholesterol (mg/dL)	159 ± 35.7	159.3 ± 36.4	170.6 ± 35.8	0.09
Triglycerides (mg/dL)	123 (80, 162)	121 (91, 181)	117 (81, 153)	0.69
HDL-C (mg/dL)	47.4 ± 11	46.6 ± 12.1	50.2 ± 15.9	0.26
LDL-C (mg/dL)	93.6 ± 32.9	90.5 ± 32.7	101.4 ± 33.4	0.28
eGFR (mL/min/1.73 m^2^)	82.2 ± 16	78.1 ± 14.9	67.2 ± 19.8	<0.01
Diabetic nephropathy, n (%)				0.02
No albuminuria	56 (37.6)	53 (35.6)	40 (26.8)	
Microalbuminuria	11 (28.9)	10 (26.3)	17 (44.7)	
Overt proteinuria	2 (22.2)	1 (11.1)	6 (66.7)	
HOMA-IR	2.4 (1.7, 4.5)	2.9 (2.2, 4.8)	2.7 (1.9, 3.9)	0.97
hsCRP (mg/dL)	0.08 (0.05, 0.18)	0.09 (0.04, 0.16)	0.09 (0.05, 0.22)	0.68
Mean CIMT (mm)	0.58 ± 0.11	0.63 ± 0.16	0.75 ± 0.14	0.03
Mean PWV (cm/sec)	1370 ± 205	1587 ± 537	1752 ± 341	<0.01
Mean ABI	1.14 ± 0.05	1.14 ± 0.07	1.14 ± 0.08	0.74

Data are shown as mean ± SD, median (interquartile range) or number (percentages).

PP: pulse pressure; SBP: systolic blood pressure; SBP: systolic blood pressure; DBP: diastolic blood pressure; BMI: body mass index; DM: diabetes mellitus; HbA1c: hemoglobin A1c; HDL: high density lipoprotein; LDL: low density lipoprotein; eGFR: estimated glomerular filtration rate; HOMA-IR: homeostasis model assessment-insulin resistance; hsCRP: high-sensitivity C-reactive protein; CIMT: carotid intima-media thickness; baPWV: brachial-ankle pulse wave velocity; ABI: ankle-brachial index.

### Prevalence of diabetic microvascular complications according to the tertile levels of central PP or brachial PP

**Figure 2:**
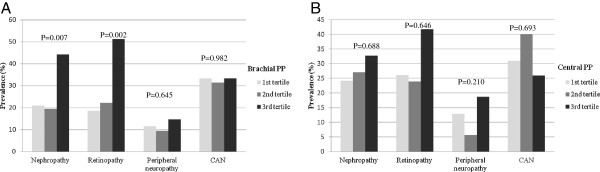
**Prevalence of diabetic microangiopathies according to the tertile levels of the PP. (A)** Brachial PP. **(B)** Central PP.

Comparisons of the prevalence of diabetic microvascular complications according to the tertile levels of central PP or brachial PP are shown in Figure 2. The prevalence of nephropathy and retinopathy according to the brachial PP tertiles significantly increased (21% vs 20% vs 44%, p = 0.006; 19% vs 22% vs 51%, p = 0.002, respectively). The prevalence of DPN and CAN did not show significant differences according to the brachial PP tertiles. The prevalence of diabetic microvascular complications did not differ across central PP tertiles.

### Differences of parameters of BP according to the presence of each diabetic microvascular complications

Differences of several parameters of BP according to the presence or absence of microvascular complications are presented in Table 5. Significantly increased levels of brachial SBP and brachial PP were detected in patients with DN compared to those without DN (p = 0.02 and p = 0.01, respectively). In patients with DR, brachial SBP and brachial PP were significantly higher than in those without DR (p = 0.02 and p = 0.03, respectively). The levels of central BP were not different between in patients with DN or DR and those without DN or DR.

**Table 5:** Blood pressure according to the presence of each microangiopathies

	**DR**			**DN**			**DPN**		
	** (−)**	** (+)**	** p**	** (−)**	** (+)**	** p**	** (−)**	** (+)**	** p**
Central SBP	119.5 ± 15.7	123.6 ± 20	0.22	120.4 ± 14.6	123.9 ± 17.8	0.20	123.2 ± 13.6	121.5 ± 21.3	0.73
Central PP	47.7 ± 11.4	53 ± 17.4	0.08	47.8 ± 11.8	51.5 ± 13.9	0.09	49.8 ± 11.4	52 ± 15.3	0.53
Brachial SBP	117.8 ± 13.6	124.7 ± 17	0.02	119.2 ± 13.9	125 ± 15.7	0.02	121.6 ± 13	122 ± 20.6	0.93
Brachial PP	46 ± 8.9	54.2 ± 15	0.03	46.6 ± 10.1	52.6 ± 13.1	0.01	48.3 ± 10.2	52.5 ± 15.1	0.21

Data are shown as mean ± SD.

DR: diabetic retinopathy; DN: diabetic nephropathy; DPN: diabetic peripheral neuropathy.

### Multivariate logistic regression analysis for the relationship of central or brachial blood pressure with presence of diabetic retinopathy/nephropathy

Clinical variables showing significantly different values between the presence or absence of DR/DN were examined by t-test (data not shown). Total cholesterol, LDL-cholesterol, eGFR and mean baPWV levels were significantly different according to the presence or absence of DN. HbA1_C_, duration of DM and mean baPWV levels were significantly different according to the presence or absence of DR. After adjustment for these variables, age and gender, multivariate logistic regression analysis was done to examine the relationship of central or brachial BP with the presence of DR/DN (Table 6). In multivariate analysis, only brachial PP among all BP components was significantly associated with the presence of DR/DN. An increased brachial PP independently increased the odds for the presence of diabetic nephropathy and retinopathy. The relative risks for the presence of DR were 1.2 and 4.6 for the brachial PP tertiles 2 and 3 when compared with the first tertile (p = 0.003). Also, the relative risks for the presence of DN were 1.02 and 3.0 for the brachial PP 2nd, 3rd tertiles when compared with first tertile (p = 0.01). Higher HbA1_C_ and longer duration of DM independently increased the odds for the presence of DR (Table 6).

**Table 6:** Multivariate logistic regression analysis with presence or absence of diabetic nephropathy/retinopathy as the dependent variable

**(1) diabetic retinopathy as dependent variable**		
**Independent variable**	**Odds ratio (95% CI)**	**P-value**
Central SBP		0.65
1st tertile	1	
2nd tertile	1.50 (0.60-3.77)	0.39
3rd tertile	1.44 (0.56-3.73)	0.45
Central PP		0.18
1st tertile	1	
2nd tertile	0.89 (0.35-2.29)	0.81
3rd tertile	2.02 (0.80-5.15)	0.14
Brachial PP		0.003
1st tertile	1	
2nd tertile	1.21 (0.44-3.54)	0.67
3rd tertile	4.59 (1.72-12.27)	0.002
Gender (Male)	1.85 (0.85-4.03)	0.12
Age	1.03 (0.99-1.07)	0.12
mean baPWV	1.01 (0.99-1.01)	0.91
HbA1C	1.85 (1.27-2.71)	0.002
Duration of DM	1.19 (1.09-1.30)	0.001
**(2) diabetic nephropathy as dependent variable**		
**Independent variable**	**Odds ratio (95% CI)**	**P-value**
Central SBP		0.69
1st tertile	1	
2nd tertile	1.28 (0.50-3.0)	0.57
3rd tertile	1.45 (0.60-3.40)	0.39
Central PP		0.61
1st tertile	1	
2nd tertile	1.17 (0.52-2.64)	0.71
3rd tertile	1.52 (0.66-3.50)	0.33
Brachial PP		0.01
1st tertile	1	
2nd tertile	1.02 (0.38-2.26)	0.86
3rd tertile	3.0 (1.32-6.80)	0.01
Age	1.02 (0.99-1.05)	0.25
Gender (Male)	1.11 (0.56-2.22)	0.76
mean baPWV	1.01 (0.99-1.01)	0.14
Total cholesterol	1.03(0.99-1.06)	0.18
LDL-cholesterol	0.98 (0.94-1.02)	0.35

SBP: systolic blood pressure; PP: pulse pressure; baPWV: brachial-ankle pulse wave velocity; LDL: low density lipoprotein; HbA1c: hemoglobin A1c.

## Discussion

To the best of our knowledge, our study is the first to report associations between central versus brachial BP with all diabetic microvascular complications as well as carotid atherosclerosis.

The main finding of this study is that central BP and brachial BP show very strong agreement. Nevertheless, the higher brachial PP levels are associated with increased probability for the presence of diabetic microvascular complications and are more powerful than central BP in relation to DR and DN. But, to the contrary, central BP levels rather than brachial BP are correlated with surrogate marker of macrovascular complications.

Peripheral (brachial) BP is an essential parameter for the evaluation and management of BP and remains the standard reference. On the other hand, there is increasing evidence that measurement of central BP, reflecting ascending aortic BP, is more strongly correlated to CVD or TOD than brachial BP levels [4,25]. Although superiority of central BP than brachial BP in relation to CVD or TOD has been suggested in several studies, measurement of central BP in real practice is not easy. Therefore, it is meaningful to examine the relationship or concordance between central BP and brachial BP. In this study, agreement between central BP and brachial BP was very strong as evidenced by the concordance correlation coefficient > 0.8. High concordance values may indicate little difference between two BP components is present and these two can be interchangeable.

Few studies have evaluated whether central and brachial BP are associated differently with carotid atherosclerosis and microvascular complications in patients with T2DM. The Strong Heart Study revealed that central PP was more strongly related to CIMT and plaque score than was brachial PP in 3520 population (including diabetes in 46.5% of women and 38.1% of men) [4]. Central pressure augmentation and aortic SBP, but not brachial SBP, were age-independent determinants of CIMT in another study [7]. To the contrary, whereas the superiority of central BP relative to brachial BP in terms of its association with TOD such as cardiac hypertrophy has been reported, central and brachial BP levels are not reportedly different in relation with cardiac hypertrophy [26]. In a recent meta-analysis of 11 longitudinal studies, the relative risk of any CV event was 1.088 ([1.040-1.139], n = 3285) for an increase of central SBP by 10 mmHg and 1.137 ([1.063-1.215], n = 4778) for an increase of central PP by 10 mmHg, but neither the RR associated with higher central SBP nor the RR associated with higher central PP differed significantly from the relative risks associated with its brachial counterparts, respectively [27].

The present study revealed that central PP is significantly correlated with CIMT and brachial PP values also showed positive, but weaker, correlation than central PP. The levels of mean CIMT demonstrated an increasing trend as the levels of central PP or brachial PP increased. However, measures of central or brachial SBP/DBP were not correlated with carotid atherosclerosis. In contrast to this our study, Westerbacka et al. reported measures of central SBP correlate with CIMT [7]. One report showed that central SBP predicted CV mortality independently of brachial SBP and traditional cardiovascular risk factors (Hazard ratio per 10 mmHg increase in central SBP: 1.34 [1.107-1.612], whereas central PP did not predict CV mortality independently of brachial PP and traditional CV risk factors in 1272 Chinese people recruited from the community [28].

PP, the arithmetic difference between systolic and diastolic BP, has been reported as a potent predictor for CVD [29]. Several studies pointed out that a CV risk in subjects with wide PP increased with the presence of diabetes [9]. In addition, PP is increased in patients with intima-media thickening [11]. In our study, brachial PP was significantly correlated with age, duration of diabetes, baPWV and CIMT. Also, central PP was correlated with age, estimated glomerular filtration ratio (eGFR), ABI, baPWV and CIMT. Especially, central or brachial PP, but not central or brachial SBP/DBP, was presently associated with carotid atherosclerosis. However, the majority of previous studies did not compare the brachial PP and central PP in relation to CV risk factors. Associations and clinical values of several components of BP such as SBP, DBP, mean BP and PP with CVD have been studied extensively. However, it is not definitively identified whether one of these measures is more strongly associated with CVD than the other. Moreover, the answer to the question of whether central BP provides value over and above peripheral BP in relation to CVD is still open.

Central BP can be directly measured only using a pressure sensor or catheter inserted into the aorta. This procedure is invasive and can lead to complications. Recently, central BP has been evaluated noninvasively by mathematically transforming the radial artery pulse waveform to the aortic pulse waveform [15,16]. However, the clinical significance of central BP, which can be measured easily by automated applanation tonometry, has not been fully elucidated. More data are needed to establish and differentiate the clinical utility of central BP using automated applanation tonometry or brachial BP as a surrogate marker in predicting CV events in T2DM.

To our knowledge, no study has examined the relative importance of central and brachial BP in their relations to microvascular complications in patients with T2DM. This present study examined the relationship between central BP, brachial BP and all microvascular complications. We established good association higher brachial PP levels and increased probability for the presence of diabetic nephropathy and retinopathy. However, our study showed that no associations of any central BP components or brachial BP components with CAN and DPN. In agreement of our study, Knudsen et al. reported that in 80 patients with T2DM, brachial PP is associated with DR and DN [12]. Also, in another study, brachial PP was reported as an important risk factor for eGFR decline and incident chronic kidney disease over a 5-year period, especially in patients with T2DM [13]. In contrast to our study, brachial PP was not a risk factor for DN in T1DM [14]. One of the possible explanation for association PP and microvascular complications such as DN and DR is that elevated PP is associated with endothelial activation and pertubation in patients with T2DM. Endothelial dysfunction could represent a pathophysiological link between these wide PP and the development of microvascular complications in T2DM [30]. However, the reasons are not clear why central PP is not associated with any diabetic microvascular complications. Prospective data on the predictive value of central BP for microvascular complications, such as renal outcome and retinal vascular impairment in diabetic patients, are currently lacking. The ongoing LOD-DIABETES study is expected to answer this question [31]. Also, it is not clear the reason why brachial PP is not associated with DPN and CAN. A possible explanation can be suggested. Regarding to the pathogenesis of DPN, several important mechanisms, such as glycemic control and duration of diabetes, have been related. Although roles of CV risk factors such as hypertension have been proposed, the effect of BP on pathogenesis of DPN may be not more prominent than other traditional risk factors of DPN [32].

Several limitations of our study should be addressed. First, due to the cross-sectional design, we cannot determine the causative relationship between brachial PP and diabetic microvascular complications, DN or DR. Prospective studies are required to address this important question. Second, because our study population included individuals who received the examination for diabetic complications, some characteristics of the present study population may be substantially different from other populations that did not perform complication study. Therefore, the generalizability of our study may be limited. Third, the present study included a small numbers of subjects. A larger number of patients should be analyzed for the confirmation of our results. Fourth, information for central BP is that derived from automated radial artery tonometry. Although the clinical significance of central BP by automated applanation tonometry, has not been fully elucidated, many studies revealed that central BP from radial artery automated tonometry showed excellent correlation with direct measured central BP [33]. However, our study is meaningful in that this is the first study for the evaluation of relationships between central versus brachial BP with all diabetic microvascular complications as well as vascular stiffness and carotid atherosclerosis in patients with T2DM.

## Conclusions

In conclusion, this study showed that agreement of central BP and brachial BP was very strong. Brachial BP are associated with presence of microvascular complications such as DR/DN than central BP. On the other hand, central BP levels rather than brachial BP are correlated with surrogate marker of macrovascular complication. However, further prospective studies are needed to evaluate the superiority or difference of central BP versus brachial BP in respective of associations with development of micro-and macrovascular complications in T2DM.

## Competing interests

The authors declare that they have no competing interests.

## Authors’ contributions

Study design: CHJ, JOM. Data collection: CHJ, SHJ, BYK. Data analysis: CHJ, SHJ, JOM. Writing the first draft: CHJ, KJK, BYK, JOM. Data interpretation, discussion and preparation of the final manuscript: CHJ, SHJ, KJK, BYK, CHK, SKK, JOM. All authors read and approved the final manuscript.

## Authors’ information

Chan-Hee Jung and Sang-Hee Jung both should be considered as first authors.

## Pre-publication history

The pre-publication history for this paper can be accessed here:

http://www.biomedcentral.com/1471-2261/14/23/prepub
